# Screen time, sleep disturbances, and dental caries in adolescents and young children: an emerging public health concern

**DOI:** 10.3389/froh.2026.1871745

**Published:** 2026-09-16

**Authors:** Saurabh RamBihariLal Shrivastava, Prateek Sudhakar Bobhate, Monal Kukde, Adeeba Siddique, Shubhada Gade

**Affiliations:** 1 Department of Community Medicine, Datta Meghe Medical College, Datta Meghe Institute of Higher Education and Research, Nagpur, Maharashtra, India; 2 Dr. D. Y. Patil School of Allied Health Sciences, Dr. D. Y. Patil Vidyapeeth, Pune (Deemed to be University), Pimpri, Pune, Maharashtra, India; 3 Department of Community Medicine, All India Institute of Medical Sciences, Vijaypur, Jammu, India; 4 Department of Dentistry, Datta Meghe Medical College, Datta Meghe Institute of Higher Education and Research, Nagpur, Maharashtra, India; 5 Department of Physiology, Datta Meghe Medical College, Datta Meghe Institute of Higher Education and Research, Nagpur, Maharashtra, India

**Keywords:** adolescents, dental caries, oral health, screen time, sleep, sugary foods

## Abstract

Dental caries is one of the leading morbidities affecting adolescents globally, with a significant proportion of adolescents continuing to experience untreated decay, pain, and functional restrictions. Increased screen time has been associated with poor oral health outcomes in both adolescent and pediatric populations, although direct evidence in adolescents is limited. Current evidence suggests an association between sleep deprivation and dental caries, although the underlying mechanisms remain incompletely understood. There is an urgent need to adopt a comprehensive set of interventions to minimize the magnitude of this problem. In conclusion, screen time, sleep deprivation, and dental caries are closely associated and may interact through several behavioral and biological pathways. Protecting adolescent health will require concerted efforts to promote device-free bedtimes, regular sleep schedules, and twice-daily fluoride brushing.

## 1 Introduction

Dental caries is one of the leading morbidities affecting adolescents globally, with a significant proportion of adolescents continuing to experience untreated decay, pain, and functional restrictions (1–3). A cross-sectional survey conducted among 11,351 adolescents in a province in China revealed that 44.6% of adolescents had dental caries (1). A systematic review from Nigeria found that 23% of adolescents had dental caries, with poor oral hygiene, consumption of refined carbohydrates, and poor utilization of dental services being the key driving forces (2). A study of children and adolescents with special healthcare needs in India reported a high prevalence of dental caries and poor oral hygiene, highlighting persistent gaps in the availability and use of preventive services (3).

## 2 Screen time and sleep deprivation in adolescents

Since the COVID-19 pandemic, a meta-analysis of 46 studies revealed an average increase of 84 min/day (52%) among the youth (4). A scoping review concluded that smartphones and handheld devices are now the most common sources of adolescent screen use, replacing televisions and desktops (5). The available literature suggests that sleep deprivation is quite prevalent among adolescents, with many adolescents not sleeping for the recommended 8–10 h of night sleep (6). A cross-sectional study performed among high school students in Iran revealed that social media applications accounted for the maximum share of screen time, while screen time was positively associated with impairment of sleep quality, daily dysfunction, and a decrease in sleep duration (7). In a prospective cohort study that followed 9,398 early adolescents for three years, researchers found that late-night screen use significantly contributed to sleep deprivation (8).

## 3 Screen time and oral health

Increased screen time has been associated with poor oral health outcomes in both adolescent and pediatric populations, although direct evidence in adolescents is limited (9–12). The results of a cross-sectional study conducted among participants aged 8–14 years (including older children and early adolescents) reported that screen time ≥2 h/day was associated with significantly higher decayed, missing, and filled teeth (DMFT) scores than those with lower screen exposure (9). Furthermore, the study found that children with higher screen time had significantly higher plaque index scores (reflecting poorer daily oral hygiene and more bacterial deposition over teeth) and higher gingival index values (i.e., high incidence of gum inflammation and early periodontal problems) (9). Another national survey conducted in Korea revealed a statistically significant association between longer smartphone screen time and self-reported caries symptoms among adolescents, highlighting adverse oral health outcomes among adolescents with digital overuse (10).

Poor oral health outcomes in adolescents with excessive screen time may be partly explained by more frequent consumption of sugary snacks and beverages, which have been associated with dental caries risk (viz., augments demineralization of enamel and cavity formation) (9). A cross-sectional observational study conducted among 100 children revealed that prolonged digital engagement often coincided with repeated snack consumption, a behavior that may increase the frequency of exposure of teeth to fermentable carbohydrates (11). Although these findings provide useful insights, they should be generalized to adolescents with caution because behavioral patterns, dietary habits, and use of digital devices vary across developmental stages. In addition, extended screen time in the evenings has been associated with delayed bedtime routines, which may indirectly affect oral hygiene practices, as reported in a cross-sectional study conducted in Turkey (12).

Furthermore, long periods of uninterrupted screen engagement are often associated with reduced water intake and greater mouth dryness, which may decrease the buffering effect of saliva and increase susceptibility to dental caries (11). Apart from increased exposure to cariogenic foods, prolonged screen engagement might encourage sedentary behavior and frequent, unstructured snacking. These eating practices increase the frequency of acid attacks on the tooth enamel by prolonging the exposure to fermentable carbohydrates. Collectively, these behavioral changes may create an oral environment that favors demineralization and the progression of dental caries (9–12).

Collectively, these findings from child- and adolescent-based studies indicate that excessive digital exposure is associated with poor diet quality, more plaque, gum problems, and a higher burden of dental caries; however, the evidence obtained exclusively from adolescents remains comparatively limited (9, 10). Despite these observations, the reported evidence must be interpreted with caution, as most studies assessing the relationship between screen time and oral health are cross-sectional, limiting the ability to establish a temporal or causal relationship. Moreover, screen time often coexists with other behavioral and socioeconomic factors, such as dietary habits, physical inactivity, parental supervision, household income, and oral hygiene practices, which may confound these associations. In addition, variations in the measurement of screen exposure, definitions of excessive screen time, and assessments of oral health outcomes can significantly contribute to heterogeneity across studies.

## 4 Sleep deprivation and dental caries

The available literature, derived from studies involving both adolescents and younger children, suggests an association between sleep deprivation and dental caries, although the underlying mechanisms remain incompletely understood (13–19). A nationally representative study conducted in the United States revealed that individuals who slept less than 7 h per night were more likely to have dental caries, suggesting that sleep duration was independently associated with dental caries after adjustment for measured covariates (13). Similarly, longer sleep duration is associated with a lower prevalence of dental caries, suggesting a dose–response association between adequate sleep and better oral health (13). A systematic review concluded that children with irregular or late bedtimes had a significantly higher risk of developing dental caries (14). The results of a narrative review indicated that disturbed sleep and disruption of circadian rhythm have been proposed as factors that may influence salivary production, which is essential for acid neutralization and enamel remineralization (15).

Moreover, sleep disturbances can affect the antimicrobial and buffering properties of saliva, thereby weakening the natural defense against cariogenic bacteria (15). Sleep disturbances may influence oral health through various interconnected biological mechanisms. For instance, circadian rhythm disruption has been associated with alterations in salivary secretion, composition, and buffering capacity, which may alter the oral microbiome environment and augment susceptibility to oral diseases (16). Inadequate sleep has also been linked to changes in immune regulation and inflammatory responses, which can alter the oral microbiome. Another scoping review reported that sleep deprivation is linked with appetite dysregulation and a preference for high-sugar foods (viz., frequent intake of sugary snacks and beverages, which are recognized risk factors for dental caries), and that inadequate sleep is linked with a wide spectrum of oral problems, extending beyond dental caries to periodontal and general oral morbidities (17). Moreover, sleep deprivation has been linked to a higher likelihood of skipping nighttime brushing or flossing due to tiredness, which allows plaque and bacterial activity overnight (17).

A recent analytical study revealed that irregular sleep patterns can reduce immune efficiency, which may create conditions favorable for bacterial proliferation and the quick progression of dental caries (18). Conversely, dental diseases, such as caries, can also contribute to sleep disturbances through pain and discomfort, creating a cycle in which poor sleep and oral diseases may influence each other through a potentially bidirectional relationship (19). Although the available literature suggests a positive association between inadequate sleep and dental caries, we must not overlook methodological constraints, as most evidence comes from observational studies that are susceptible to confounding factors (such as dietary practices, socioeconomic status, and access to dental care) (15–17). In addition, the studies vary according to the criteria set for sleep duration, sleep quality, and dental caries, making the comparison between these studies difficult (16–19). These limitations highlight the need for systematically planned longitudinal and interventional studies to gain deeper insights into the nature and direction of these reported associations.

At this juncture, it is important to acknowledge that screen time, sleep disturbances, and dental caries constitute an interconnected behavioral and biological framework, rather than acting as independent risk factors. Excessive screen use may contribute to delayed sleep, unhealthy dietary behaviors, and poor oral hygiene practices. In addition, insufficient sleep may further affect appetite regulation, salivary physiology, immune function, and decision-making. As these interacting mechanisms cumulatively enhance susceptibility to dental caries, there is an urgent need to adopt integrated preventive approaches that target multiple lifestyle behaviors simultaneously.

## 5 Preventive strategies

Given the potential association between screen time and sleep disturbances, as well as the burden of dental caries and other oral problems among adolescents, and the bidirectional relationship between these issues, there is an urgent need to adopt a comprehensive set of interventions to minimize the magnitude of the problem (11, 17, 20–22). To begin with, at the individual and family levels, there is an immense need to limit recreational screen time to age-appropriate durations, as this may help reduce sedentary snacking and bedtime delays, and can also discourage unhealthy routines, which can indirectly augment the risk of caries (17). Not allowing screen time before bedtime may support sleep by improving the release of melatonin and the quick onset of sleep, which may improve sleep duration and potentially benefit oral health (20). Furthermore, regular sleep schedules should be maintained, including weekends, as this will support the circadian rhythm, which will eventually aid in the regulation of salivary flow and the recovery of oral tissues (21).

Brushing in the morning and before bed with fluoride toothpaste remains one of the most effective approaches to preventing caries, particularly among individuals with suboptimal oral hygiene (22). In addition, avoiding sugary snacks and soft drinks during screen use or gaming can play a vital role in preventing tooth enamel decay (22). In addition, encouraging water intake during long screen sessions can reduce dry mouth and even aid saliva's buffering action, which protects against acid damage and bacterial growth (11). The next set of interventions is related to dental healthcare, including scheduling regular dental check-ups every 6 months for the early detection of oral lesions, regular hygiene counseling, and identification of lifestyle risks (22). It is important to integrate sleep screening into dental visits. This includes asking questions about bedtime habits, snoring, late-night device use, and sleep duration to identify hidden contributors to oral disease (17).

Family-centered counseling may promote improved adherence to bedtime brushing, reduce nighttime screen exposure, and encourage healthy dietary habits (13). Children and adolescents with behavioral risk factors can be administered preventive fluoride therapies and fissure sealants to reduce the incidence of caries (22). In this regard, schools have a crucial role to play, wherein school-based oral health education can be linked with digital lifestyle awareness, including an explanation of the relationship between screen time, poor sleep, sugary snacking, and dental cavities (22). Furthermore, healthy school schedules can be encouraged to support adequate sleep with sufficient time for rest (17). Moreover, there should be restrictions on the sale of sugary beverages and junk foods within school premises, as this will reduce the frequent intake of sugar during the day (22). Finally, integrating public health guidance that targets adolescent screen time, sleep hygiene, and oral health can s address these interconnected risk factors more effectively than isolated initiatives (17, 22).

## 6 Implications for the future

Future research should move beyond examining screen time, sleep deprivation, and dental caries as isolated entities and explore their interconnected pathways and mechanisms. Furthermore, proposed future research must consider methodological limitations, including reliance on cross-sectional studies, variability in the definition of screen exposure and sleep disturbances, lack of adjustment for potential confounders, and heterogeneity in outcome assessment. Future research should also explore the complex behavioral and biological pathways linking excessive screen use, sleep disturbances, and oral health. The recent literature suggests that excessive digital engagement is expected to impact oral health through different behavioral and biological methods, including sleep disruption, changed dietary habits, poor oral hygiene practices, and psychosocial stress. From a public health perspective, these factors must be acknowledged and considered while understanding the broader digital lifestyle environment that affects adolescent health.

We strongly advocate that future oral health promotion interventions should include digital wellness and sleep hygiene components in addition to conventional preventive measures. Families, schools, and healthcare delivery systems must adopt integrated approaches that cumulatively target screening habits, sleep behaviors, dietary practices, and oral hygiene. In addition, prospective studies should be conducted to establish causality and identify effective intervention approaches. As digital technology has become an integral part of almost every aspect of adolescent lives, addressing interconnected determinants may improve both oral and general health outcomes. An important limitation of the existing evidence is that multiple studies have been conducted in young children, but these findings should be extrapolated to adolescents with caution. However, considering the unique developmental, psychosocial, and digital engagement habits of adolescents, future research should specifically examine these relationships in adolescent populations.

## 7 Conclusion

Screen time, sleep deprivation, and dental caries are closely associated and may interact through multiple behavioral and biological pathways. Protecting adolescent health will require concerted efforts to promote device-free bedtimes, maintain regular sleep schedules, and twice-daily fluoride brushing.
